# Quantifying the Diagnosis and Survival of Early Onset Bowel Cancer Among First Nations Peoples in Queensland, Australia

**DOI:** 10.1002/cam4.70821

**Published:** 2025-03-21

**Authors:** Tsegaw Amare Baykeda, Shafkat Jahan, Kirsten Howard, Rakhee Raghunandan, Gail Garvey

**Affiliations:** ^1^ School of Public Health, Faculty of Health, Medicine and Behavioural Science The University of Queensland Brisbane Australia; ^2^ The Leeder Centre for Health Policy, Economics and Data, School of Public Health, Faculty of Medicine and Health The University of Sydney Sydney Australia

**Keywords:** aboriginal and Torres Strait islander peoples, bowel cancer, early onset bowel cancer, First Nations Indigenous Australians, young‐onset bowel cancer

## Abstract

**Introduction:**

The incidence of early‐onset bowel cancer (EOBC) is increasing in Australia and globally. However, the burden of EOBC among First Nations Australians is rarely determined. This study aimed to quantify the diagnosis and survival rates of EOBC among First Nations Peoples in Queensland, Australia.

**Methods:**

CancerCostMod, a linked administrative dataset of patients diagnosed with cancer in Queensland from 1st July 2011 to 30th June 2015, was used. EOBC was defined as a diagnosis of bowel cancer (i.e., colon, rectosigmoid, or rectal cancer) at 18–49 years of age. A multivariable logistic regression analysis was employed to determine the association of Indigenous status and other factors with a diagnosis of EOBC. Five‐year survival rates were used to estimate the survival rate.

**Results:**

Of 11,702 bowel cancer cases, 9.2% (95% CI: 8.7%–9.7%) were EOBC, with 19% among First Nations peoples and 9% among Non‐First Nations. First Nations Australians had 2.6 times the odds of EOBC diagnosis (95% CI: 1.7–4.0) compared with Non‐First Nations Australians. Overall, EOBC patients showed a significantly higher 5‐year survival rate of 77% compared with 60% for late‐onset bowel cancer patients. However, First Nations EOBC patients showed a lower 5‐year survival rate (73%) than Non‐First Nations EOBC patients (77%).

**Conclusion:**

First Nations Australians have more than double the diagnosis rates and lower 5‐year survival for EOBC compared to Non‐First Nations. Whilst the recent lowering of the age eligibility for the National Bowel Cancer Screening Program is a beneficial strategy to address the increasing incidence of EOBC, special consideration should be given to addressing the higher diagnosis rates and lower survival among First Nations Australians. This study raises the potential for further lowering the age eligibility for First Nations Australians to ensure younger First Nations Australians can access screening for earlier detection, thereby improving their survival from bowel cancer.

## 1 Introduction

Bowel (colorectal) cancer is typically associated with older age [1], with a median age at diagnosis of 70 years in Australia [2]. However, individuals younger than 50 years are increasingly diagnosed with bowel cancer, and this diagnosis is referred to as early‐onset bowel cancer (EOBC) [3, 4, 5, 6].

Globally, the incidence of EOBC has more than doubled between 1990 and 2019, rising from 95,737 to 226,782 [7]. In 2022, 10% (184,709) of all new bowel cancer cases were diagnosed in adults under 50 years old [8]. Bowel cancer is the most common cancer in Australia, with 1716 cases (11% of all cases) diagnosed among those under 50 years old in 2019 [8, 9, 10]. Over the past three decades (1989–2019), the diagnosis of bowel cancer in Australians under 50 years old has increased by 142%, compared with a 76% increase across all age groups [2].

Despite better survival rates compared to older adults [9, 10], EOBC has been associated with early menopause in women [11], body image changes [12], and sexual health issues [13]. Additionally, EOBC cases are often more aggressive and diagnosed at advanced stages [14], as National Bowel Cancer Screening Programs (NBCSP) typically start screening at the age of 50 years [15, 16, 17] and miss younger individuals. However, as of July 1, 2024, eligibility for the NBCSP in Australia has been lowered to include individuals aged 45 years [18].

Worldwide studies indicate a disparity in the incidence and survival of EOBC between Indigenous and Non‐Indigenous peoples. In the USA, EOBC incidence is 8%–21% higher among American Indians/Alaskan Natives than among Whites [19, 20]. Additionally, from 2009 to 2018, the incidence of EOBC in American Indians/Alaskan Natives increased twice as fast as that in Whites [21]. In New Zealand, the incidence of EOBC among Māori people increased by 36% compared with 26% for the general population between 2000 and 2020 [22]. Moreover, in Australia's Northern Territory, Aboriginal and/or Torres Strait Islander people, hereafter respectfully referred to as First Nations Australians, faced twice the mortality risk from early‐onset gastrointestinal cancer (including bowel cancer) compared with Non‐First Nations Australians [23]. First Nations Australians also have a lower overall 5‐year survival rate from bowel cancer of 63% compared with 70% for Non‐First Nations Australians [24].

First Nations Australians refers to people who have identified themselves as being of Aboriginal and/or Torres Strait Islander origin. First Nations Australians represented 3.8% (983,700 people) of the total Australian population (26,638,544 people) in 2021 [25]. Almost two‐thirds of First Nations Australians live in New South Wales (34%) and Queensland (29%) while the Northern Territory had the highest percentage of First Nations peoples within its population (31%; 76,487 people) [25, 26]. First Nations people are more likely to live in urban and regional areas than in remote areas. However, the percentage of the total population who are First Nations tends to be higher in more remote areas [26].

Various social determinants of health, along with historical issues such as colonisation and discrimination, have influenced the health outcomes of First Nations Australians [27, 28]. The reasons for the increased diagnosis and lower survival of bowel cancer among First Nations peoples are complex and multifactorial. These include sociodemographic factors, healthcare access, treatment and prevention differences, dietary habits, and genetic influences. However, the disparity in the diagnosis of EOBC and its survival among First Nations and Non‐First Nations Australians remains undetermined.

Understanding potential reasons for this disparity will help design targeted interventions to improve outcomes from bowel cancer among First Nations Australians. Thus, contributing to more equitable healthcare policies in Australia, aligning with achieving Closing‐the‐Gap targets, specifically, “Aboriginal and Torres Strait Islander people enjoy long and healthy lives” [29]. Therefore, this study aimed to quantify and compare the diagnosis and survival of EOBC among First Nations and Non‐First Nations peoples in Queensland, Australia.

## 2 Materials and Methods

### 2.1 Data Sources

The CancerCostMod, a linked administrative dataset, was used in this study. The development of the CancerCostMod dataset is published elsewhere [30, 31]. Briefly, CancerCostMod includes 106,749 patients diagnosed with cancer (except those with non‐melanoma skin cancer) between July 1, 2011 and June 30, 2015 in Queensland. CancerCostMod also includes linked data for these patients until June 30, 2018, ensuring at least 3 years of follow‐up for each patient [32].

Each patient's demographic and cancer data were obtained from the Queensland Cancer Registry (QCR) and linked to records in the Queensland Health Admitted Patient Data Collection (QHAPDC), Queensland Health Emergency Department Information Systems (EDIS), Medicare Benefits Schedule (MBS), and Pharmaceutical Benefits Scheme (PBS) from July 1, 2011 to June 30, 2018. This study used the linked QCR and QHAPDC data from July 1, 2011 to June 30, 2015 to explore early‐onset bowel cancer and survival among First Nations Australians.

The QCR data include age, gender, marital status, Indigenous status, patient's postcode, date of diagnosis, financial year of diagnosis, date of death, cause of death site code, histological grading, and cancer type site code. The QHAPDC includes details about hospital episodes and diagnoses. The 10th International Classification of Diseases (ICD‐10) site codes from QCR were used to identify bowel cancer patients by summing cases of colon cancer (ICD‐10 code C18), rectosigmoid cancer (C19), and rectal cancer (C20) and the diagnoses information from QHAPDC was used to calculate the Charlson Comorbidity Index (CCI).

### 2.2 Study Variables

The outcome variables of this study were the diagnosis and survival of EOBC. The independent variables include Indigenous status, sex, marital status, remoteness, Index of Relative Socioeconomic Disadvantage (IRSD) score, cancer type, the financial year of diagnosis, comorbidity, tumor grade, and vital status.

### 2.3 Operational Definitions

- EOBC was defined as 18–49 years of age at diagnosis with bowel cancer (yes/no) [4, 33].
- Bowel cancer type grouped as colon cancer (C18) or rectal cancer (C19 and C20).
- Indigenous status was classified as First Nations for Indigenous Australians (includes Aboriginal and/or Torres Strait Islander people) and Non‐First Nations for Non‐Indigenous Australians. Indigenous status is determined by an individual's self‐identification as Aboriginal only, both Aboriginal and Torres Strait Islander, or Torres Strait Islander only [34].
- IRSD was measured using the patients' postcode based on the Australian Statistical Geography Standard [35]. The Index was measured in quantiles, and we further categorised them into three groups: Quantiles 1–2: most disadvantaged, Quantile 3: and Quantiles 4–5: least disadvantaged.
- Remoteness was measured using the patients' postcode based on the Australian Statistical Geography Standard [36]. Initially, it was measured as metropolitan, inner regional, outer regional, remote, and very remote. We further regrouped the classification into metropolitan, regional (including both outer and inner regional) and remote (including both remote and very remote).
- Marital status was measured as married (includes de facto) and not married (includes divorced, separated, widowed and never married).
- The financial year of diagnosis was used instead of the calendar year of diagnosis since the records of the dataset were reported from July 1, 2011 to June 30, 2015 following the financial period of reporting.
- Comorbidity status was calculated using the Charlson Comorbidity Index (CCI), initially scoring 0, 1, 2, and 3+. We collapsed the scores into 0 (no comorbidity) and 1+ (yes comorbidity).
- Tumour grading was measured using a histological differentiation as Grade I: well‐differentiated, Grade II: moderately differentiated, Grade III: poorly differentiated, and Grade IV: undifferentiated. We further regrouped as Grades I–II (low grade) and Grades III–IV (high grade) [9].

### 2.4 Statistical Analysis

A descriptive analysis using frequency and percentage was conducted to describe the characteristics of patients diagnosed with bowel cancer. A cross‐tabulation with Pearson's chi‐square test was used to demonstrate the relationship between the characteristics of patients and their Indigenous and EOBC statuses. Additionally, the median age of diagnosis among First Nations and Non‐First Nations peoples was calculated and tested for significant variation using the non‐parametric Mann–Whitney U test, as the normality assumption failed for an independent sample *t* test.

A stepwise logistic regression analysis was employed to identify significant explanatory variables, and models with various combinations of variables were fitted. We selected the stepwise method for variable selection because it allows the data to guide the inclusion of variables without prior strong assumptions, making it particularly useful in exploratory analyses. This approach also enables us to assess how Indigenous status is associated with EOBC diagnosis adjusted for the presence of the other variables. To minimize the risk of model overfitting, we pre‐selected variables that exhibited *p* values < 0.2 in the univariate logistic regression analysis. Additionally, model performance was evaluated using *R* ^*2*^ and the Akaike Information Criterion (AIC), with higher *R* ^*2*^ and lower *AIC* values indicating a better‐fitting model.

In a stepwise logistic regression analysis, Indigenous status was adjusted with different combinations of variables from Models II–IV. Model I included Indigenous status alone associated with EOBC. In Model II, individual‐level factors were added to Model I. Model III incorporated community‐level factors into Model II, while Model IV included clinical‐level factors into Model III. Model IV was selected as the final model based on its lower AIC and higher *R* ^*2*^. In the final model, demographic variables (Indigenous status, sex, and marital status), community‐level variables (remoteness and IRSD), and clinical variables (comorbidity, cancer type, and tumour grade) were fitted. Model outputs were reported as odds ratios (ORs) and 95% confidence intervals (CIs). In all models, Indigenous status showed a significant association with EOBC, with a *p* < 0.001.

Finally, we calculated the 5‐year survival rates of the patients based on their Indigenous and EOBC statuses using the survival function. Cox proportional hazard regression models were used to calculate the adjusted hazard ratio (aHR) and 95% CI, comparing the risk of mortality among early‐onset versus late‐onset bowel cancer patients and among First Nations and Non‐First Nations patients with EOBC. For overall survival, the outcome was death from any cause, while for bowel cancer‐specific survival, the outcome was death from bowel cancer only. In both groups, patients who were alive or who died from other causes were censored at the last date of data collection, which was June 30, 2018, for those who were still alive and at the date of death for those who had died from other causes. Survival time was measured in days and reported in months, calculated from the date of diagnosis to the date of death or censoring.

### 2.5 Sensitivity Analysis

Using Pearson's chi‐square tests, we conducted a sensitivity analysis to explore how bowel cancer incidence varies between First Nations and Non‐First Nations Australians when applying different diagnosis age thresholds: 50, 45, 40, and 35 years. These findings provide epidemiological evidence that may inform policy discussions on the age eligibility for the NBCSP. Such adjustments could help address disparities in bowel cancer incidence and support earlier detection, especially among First Nations populations.

A significance level of *p* < 0.05 was used to identify significant factors in all analyses. All statistical analyses were performed using Stata 18 software.

### 2.6 Ethical Considerations

Ethical approval was obtained from the Townsville Hospital and Health Service Human Research Ethics Committee on July 15, 2016 (HREC/16/QTHS/110), the Australian Institute of Health and Welfare (AIHW) ethics Committee on July 15, 2016 (EO2017/1/343), and the University of Queensland Human Research Ethics Committee (UQHREC by 2022/HE002538). Permission to waive consent was obtained under the Public Health Act 2005. De‐identified data are securely uploaded by the AIHW to the Secure Unified Research Environment (SURE) facility, where researchers exclusively access it.

## 3 Results

### 3.1 Sociodemographic Characteristics of Bowel Cancer Patients

The study included 11,702 bowel cancer patients with a median diagnosis age of 71 years (interquartile range [IQR]: 61–79). The overall proportion of EOBC diagnosis was 9% (95% CI: 8.7%–9.7%), but it was higher in First Nations (19%) compared with Non‐First Nations Australians (9%) (*p* < 0.001). Most Non‐First Nations Australians were from metropolitan (46%) and regional (45%) areas compared with First Nations, and a higher proportion of First Nations peoples resided in remote (28%) areas compared with Non‐First Nations peoples (8%) (*p* < 0.001). A higher proportion (28%) of First Nations patients were in the most disadvantaged groups (IRSD Quantiles 1–2) compared with Non‐First Nations patients (14%), while a lower proportion of First Nations represented the least disadvantaged group (IRSD Quantiles 4–5) (50%) compared with Non‐First Nations (69%) (*p* < 0.001) (Table 1).

**TABLE 1:** Sociodemographic characteristics of bowel cancer patients in Queensland, Australia, diagnosed from July 1, 2011 to June 30, 2015 (*N* = 11,702).

Variables	All		Indigenous status				
	*N* = 11,702		First Nations		Non‐First Nations*		*p* value
			*N* = 155		*N* = 11,536		
	*N*	%	*N*	%	*N*	%	
Age at diagnosis (years)							
Median (IQR)	71 (61–79)		63 (52–72)		71 (61–79)		< 0.001
18–49	1075	9.19	30	19.35	1044	9.05	< 0.001
≥ 50	10,627	90.81	125	80.65	10, 492	90.95	
Cancer type							
Colon	8083	69.07	99	63.87	7977	69.15	0.158
Rectal	3619	30.93	56	36.13	3559	30.85	
Gender							
Male	6369	54.43	87	56.13	6277	54.41	0.670
Female	5333	45.57	68	43.87	5259	45.59	
Remoteness**							
Metropolitan	5384	46.01	51	32.90	5326	46.17	< 0.001
Regional	5252	44.88	59	38.06	8189	44.98	
Remote	999	8.54	43	27.74	956	8.29	
IRSD**							
Quantiles 1–2	1620	13.84	43	28.10	1577	13.75	< 0.001
Quantile 3	1981	16.93	33	21.57	1947	16.97	
Quantile 4–5	8033	68.65	77	50.33	7947	69.28	
Marital status							
Married	7132	60.95	73	47.10	7059	61.19	< 0.001
Not married	4570	39.05	82	52.90	4477	38.81	
Financial year of diagnosis							
2012	2955	25.25	28	18.06	2924	25.35	0.195
2013	2875	24.57	39	25.16	2833	24.56	
2014	2830	24.18	41	26.45	2789	24.18	
2015	3042	26.00	47	30.32	2990	25.92	
Comorbidity							
No	7814	66.77	100	65.36	7600	66.46	0.774
Yes	3888	33.23	53	34.64	3835	33.54	
Tumour grade							
Grades I–II	7593	64.89	103	66.45	7484	64.88	0.127
Grades III–IV	2155	18.42	20	12.90	2133	18.49	
Unknown	1954	16.70	32	20.65	1919	16.63	

*Note:* % is a column percentage within each variable.

Abbreviations: IQR, interquartile range; IRSD: index of relative socioeconomic disadvantage.

* Does not include “unknowns” (*n* = 11; 0.09%).

** Does not include those with missing postcode data at diagnosis (*n* = 67; 0.57%).

### 3.2 EOBC Across patients' Characteristics

The study also showed that a higher proportion of females (10%) were diagnosed with EOBC compared to males (8%) (*p* = 0.001). Additionally, 10% of rectal cancer cases were diagnosed among individuals younger than 50 years which is higher compared to colon cancer cases (9%) (*p* = 0.002) (Table 2).

**TABLE 2:** EOBC across sociodemographic characteristics of patients diagnosed between July 1, 2011 and June 30, 2015 in Queensland, Australia (*N* = 11,702).

Variables	EOBC				
	Yes		No		*p* value
	*N*	%	*N*	%	
Indigenous status					
First Nations	30	19.35	125	80.65	< 0.001
Non‐First Nations	1044	9.05	10,492	90.95	
Gender					
Male	531	8.34	5838	91.66	0.001
Female	544	10.20	4789	89.80	
Remoteness					
Metropolitan	527	9.79	4857	90.21	0.073
Regional	447	8.51	4805	91.49	
Remote	90	9.01	909	90.99	
IRSD					
Quantiles 1–2	120	7.40	1501	92.60	0.032
Quantile 3	194	9.79	1787	90.21	
Quantiles 4–5	750	9.34	7283	90.66	
Marital status					
Married	744	10.43	6388	89.57	< 0.001
Not married	331	7.24	4239	92.76	
Comorbidity					
No	787	10.07	7027	89.93	< 0.001
Yes	288	7.41	3600	92.59	
Cancer type					
Colon	697	8.62	7386	91.38	0.002
Rectal	378	10.44	3241	89.56	
Financial year of diagnosis					
2012	237	8.02	2718	91.98	0.018
2013	278	9.67	2597	90.33	
2014	291	10.28	2539	89.72	
2015	269	8.84	2773	91.16	
Tumour grade					
Grades I–II	609	8.02	6984	91.98	< 0.001
Grades III–IV	194	9.00	1961	91.00	
Unknown	272	13.92	1682	86.08	

*Note:* % is a row percentage within each category.

### 3.3 Factors Associated With the Early Onset of Bowel Cancer

First Nations Australians have 2.62 times the odds of EOBC diagnosis compared with Non‐First Nations Australians. Females have a higher likelihood of EOBC diagnosis (OR = 1.36; 95% CI: 1.19–1.55) than males. Married people have a higher likelihood of EOBC diagnosis (OR = 1.66; 95% CI: 1.44–1.91) compared to those who are not married. The odds of EOBC were lower (OR = 0.74, 95% CI: 0.64–0.85) among those with comorbidity compared with those without comorbidity. There was also a higher likelihood of early onset diagnosis of rectal cancer (OR = 1.26; 95% CI: 1.10–1.45) compared to colon cancer (Table 3).

**TABLE 3:** Stepwise logistic regression analysis of factors associated with EOBC among patients diagnosed from July 1, 2011 to June 30, 2015 in Queensland, Australia (*N* = 11,702).

Variables	Model I	Model II	Model III	Model IV
	OR (95% CI)	OR (95% CI)	OR (95% CI)	OR (95% CI)
First Nations	2.41 (1.61–3.61)*	2.60 (1.73–3.90)*	2.65 (1.75–4.02)*	2.62 (1.73–3.99)*
Female		1.36 (1.20–1.55)*	1.35 (1.19–1.55)*	1.36 (1.19–1.55)*
Married		1.61 (1.40–1.55)*	1.61 (1.40–1.85)*	1.66 (1.44–1.91)*
Regional			0.88 (0.76–1.02)	0.88 (0.76–1.01)
Remote			0.89 (0.69–1.14)	0.87 (0.68–1.12)
Quantile 1–2			0.81 (0.65–1.01)	0.81 (0.65–1.01)
Comorbidity, yes				0.74 (0.64–0.85)*
Rectal cancer				1.26 (1.10–1.45)*
Grades III and IV				1.15 (0.97–1.37)
*R* ^2^	0.0021	0.0104	0.0121	0.0246
AIC	7163.08	7107.70	7044.93	6960.30

Abbreviations: AIC: Akaike Information Criterion; OR: Odds Ratio; CI: Confidence Interval.

* *p* < 0.001.

### 3.4 Survival Difference Among Early‐Onset and Late‐Onset Bowel Cancer Patients

This study showed that 61% of all bowel cancer patients had not died by the end of the study period (July 30, 2018) with a significantly higher proportion (78%) of EOBC patients compared with late‐onset bowel cancer patients (60%). In addition, the 5‐year survival rate for early‐onset bowel cancer patients was 77% (95% CI: 74.32%–79.64%), which is higher than the 60% (95% CI: 58.65%–60.65%) survival rate among late‐onset patients. Moreover, the early‐onset patients have 49% and 34% lower all‐cause mortality and bowel‐cancer‐specific mortality rates than those of late‐onset patients, respectively (Table 4).

**TABLE 4:** Survival difference among early onset and late onset bowel cancer patients diagnosed from July 1, 2011 to June 30, 2015 in Queensland, Australia (*N* = 11,702).

Variables	Total *N* (%)	Early‐onset	Late‐onset	*p* value
	*N* = 11,702	*N* = 1075	*N* = 10,627	< 0.001
Did not die	7170 (61.27)	836 (77.77)	6334 (59.60)	< 0.001
Died due to bowel cancer	3406 (29.11)	228 (21.21)	3178 (29.90)	
Died due to other causes	1126 (9.62)	11 (1.02)	1115 (10.49)	
Median (IQR) survival (months)	46.62 (24.66–63.62)	52.50 (39.22–66.67)	45.96 (22.85–63.19)	< 0.001
5‐year survival rate (95% CI)	61.27 (60.32–62.20)	77.11 (74.32–79.64)	59.66 (58.65–60.65)	
HR (95% CI)* ^,^ ^#^	—	0.51 (0.45–0.58)	Ref	< 0.001
HR (95% CI)** ^,^ ^#^	—	0.66 (0.58–0.76)	Ref	< 0.001

* Overall survival.

** Bowel‐cancer‐specific survival; HR: hazard ratio.

^#^ Adjusted for sex, marital status, remoteness, IRSD.

### 3.5 Survival Difference Among First Nations and Non‐First Nations Australians With EOBC

In this study, a higher proportion (78%) of Non‐First Nations Australians survived EOBC than First Nations Australians (73%) (*p* = 0.556). In addition, the median survival of EOBC for First Nations Australians was shorter (50 months) compared with Non‐First Nations Australians (53 months) *(p = 0.140)*. Moreover, the 5‐year survival rate of EOBC was lower for First Nations Australians (73%; 95% CI: 53.44–85.59) compared to Non‐First Nations Australians (77%; 95% CI: 74.36–79.77) (Table 5).

**TABLE 5:** Survival difference of EOBC among First Nations and Non‐First Nations Australians diagnosed from July 1, 2011 to June 30, 2015 in Queensland, Australia (*N* = 11,702).

Variables	First Nations	Non–First Nations	*p* value
	*N* = 30	*N* = 1044	
Did not die	22 (73.33)	813 (77.87)	0.556
Died	8 (26.67)	231 (22.13)	
Median (IQR) survival (months)	50.23 (36.43–66.51)	52.50 (39.39–66.67)	0.140
5‐year survival rate (95% CI)	73.18 (53.44–85.59)	77.20 (74.36–79.77)	
HR (95% CI)* ^,^ ^#^	1.47 (0.72–2.99)	Ref	0.288
HR (95% CI)** ^,^ ^#^	1.35 (0.63–2.89)	Ref	0.432

* Overall survival.

** Bowel cancer‐specific survival; HR: hazard ratio.

# Adjusted for sex, marital status, remoteness, and IRSD.

### 3.6 Sensitivity Analysis

The sensitivity analysis, conducted using different age‐at‐diagnosis classifications for EOBC, revealed that bowel cancer diagnoses are significantly higher among First Nations Australians compared to Non‐First Nations Australians across all age groups. The analysis also showed that the difference in EOBC diagnoses between First Nations and Non‐First Nations Australians decreased as the cutoff age for diagnosis was lowered (50, 45, 40, and 35 years). The difference in the proportion of EOBC diagnoses between First Nations and Non‐First Nations Australians at a 50‐year cutoff age was 10.3% (*p* < 0.001), at 45 years 5.5% (*p* = 0.016), at 40 years 4.2% (*p* = 0.011), and at 35 years 3.9% (*p* = 0.002) (Table 6).

**TABLE 6:** Proportion of EOBC diagnosis at different ages at diagnosis among First Nations and Non‐First Nations Australians diagnosed from July 1, 2011 to June 30, 2015 in Queensland, Australia (*N* = 11,702).

Variable	Age at diagnosis	First Nations		Non‐First Nations		Diff (%)	*p* value
		*N*	%	*N*	%		
EOBC	18–49	30	19.35	1044	9.05	*10.30*	< 0.001
	50+	125	80.65	10,492	90.95		
Sensitivity analysis—1	18–44	18	11.61	701	6.08	*5.53*	0.016
	45+	137	88.39	10,835	93.92		
Sensitivity analysis—2	18–39	13	8.39	486	4.21	*4.18*	0.011
	40+	142	91.61	11,050	95.79		
Sensitivity analysis—3	18–34	10	6.45	291	2.52	*3.93*	0.002
	35+	145	93.55	11,245	97.48		

Abbreviation: Diff: difference.

## 4 Discussion

Using linked administrative data from Queensland, Australia's second‐largest state by First Nations population, this study provides important epidemiological evidence on the incidence of early‐onset bowel cancer compared to late‐onset bowel cancer. It also explored the disparities in diagnosis and survival rates of early‐onset bowel cancer between First Nations and non‐First Nations Australians. Overall, the diagnosis rate of early‐onset bowel cancer is higher among First Nations Australians than among non‐First Nations Australians. First Nations Australians diagnosed with early‐onset bowel cancer experience lower survival rates than their non‐First Nations counterparts.

In this study, 9.2% (95% CI: 8.7%–9.7%) of bowel cancer patients in Queensland were diagnosed under the age of 50. This finding is consistent with the national Australian report, which showed that 9.2% (95% CI: 9.0%–9.4%) of bowel cancer patients diagnosed between 2012 and 2015 were in individuals under 50 [2], and with the global proportion in 2022, where 9.6% (95% CI: 9.55%–9.63%) of bowel cancer patients were under the age of 50 [8]. Additionally, EOBC cases increased by 14% from 2012 to 2015 in Queensland, slightly lower than the 21% increase observed nationally during the same period [2]. This lower percentage change may be due to differences in population size, the time classification (calendar year used nationally versus financial year in this study), and age classification (18–49 years in this study vs. under 50 nationally).

Nevertheless, the 14% increase in EOBC cases from 2012 to 2015 in Queensland observed in this study is considerably higher than the 2% increase observed in late‐onset bowel cancer and the 3% increase in all‐age bowel cancer during the same period. This is consistent with the national report, which found a 21% increase in EOBC, compared to only a 3% increase in late‐onset and a 4% increase in all‐age bowel cancer during the same period [2]. Globally, EOBC cases are projected to increase by 21% from 2022 to 2050, while late‐onset cases are projected to rise by 93% and all‐age bowel cancer cases by 86% [37]. Global estimates also indicate that Australia leads in EOBC with an age‐standardised incidence rate of 18/100,000 in 2022 [37], highlighting the need for urgent policy intervention. The reasons behind the rising incidence of EOBC are not fully understood [38, 39]. However, genetic factors and increased early exposure to environmental risk factors such as a sedentary lifestyle, obesity, smoking, heavy alcohol use, and an unhealthy diet are likely associated with EOBC [39, 40, 41, 42, 43].

Consistent with other studies [9, 10, 44, 45], EOBC patients showed a significantly higher 5‐year survival rate (77%) than late‐onset patients (60%). However, higher 5‐year survival rates among those with EOBC should be interpreted with caution, as bowel cancer diagnosis in younger individuals has a better outcome than diagnosis in older individuals. Potential reasons for this finding include that younger people are generally healthier than older adults (this study also found comorbidity is significantly lower among EOBC patients) and are typically more tolerant of treatment‐related toxicities [46]. However, the higher 5‐year survival rate in EOBC does provide support for the recently introduced initiative to lower the age eligibility for the Australian NBCSP to all 45‐year‐olds. Early detection of bowel cancer is associated with better health outcomes [47]. Therefore, lowering the age eligibility for the Australian NBCSP and detecting EOBC earlier can provide patients with earlier access to treatment.

The proportion of EOBC is significantly higher in First Nations Australians, with 2.6 times the odds of EOBC diagnosis compared to Non‐First Nations Australians. This is consistent with studies conducted in the USA [19, 20, 21] and New Zealand [22], where the proportion of EOBC among Native Americans and Māori peoples was found to be significantly higher compared with those among non‐Indigenous peoples, respectively. This disparity might be due to several factors: Firstly, it might be related to a higher exposure to risk factors among First Nations peoples [48]. Additionally, the younger demographic (median age of First Nations is 24 years compared to 38.9 for Non‐First Nations) [25] and the lower life expectancy of First Nations peoples could contribute to this higher proportion. Lastly, the higher screen positivity rate among First Nations than Non‐First Nations may result in more cases being diagnosed through doctor‐recommended screenings.

This study showed that First Nations Australians with EOBC had lower survival rates for bowel cancer compared with Non‐First Nations Australians with EOBC, with a 5‐year survival rate of 73% compared with 77%, respectively. Furthermore, First Nations Australians with EOBC have a 47% and 35% higher risk of mortality from all causes and bowel cancer only than Non‐First Nations Australians with EOBC. This result is similar to that of a study conducted in the Northern Territory, where First Nations Australians showed twice the risk of mortality from early‐onset gastrointestinal cancer (including bowel cancer) compared with Non‐First Nations Peoples [23]. Limited access to healthcare and lower health service utilization among First Nations Peoples [49] might be associated with this survival difference. Because the routine screening program did not include individuals aged less than 50, diagnoses would have been made based on patients' awareness of the symptoms of bowel cancer and their corresponding early visits to their healthcare provider. Hence, First Nations Peoples might be diagnosed at an advanced stage of the disease when its survival rate is lower.

The Australian government's new policy of lowering the eligibility age for the NBCSP to 45 years could help reduce disparities in bowel cancer diagnosis and survival. Our sensitivity analysis supports this, showing that reducing the age of typical onset bowel cancer from 50 years old to include younger ages ranging from 35 to 45 years old reduces the difference in bowel cancer diagnosis between First Nations and Non‐First Nations Australians. Lowering the age eligibility further for First Nations Australians might capture more cases of bowel cancer, especially those with EOBC and help address the survival disparity noted between First Nations and Non‐First Nations Australians with EOBC. This suggested approach is similar to New Zealand's policy of lower‐age eligibility for bowel cancer screening for Māori and Pasifika (50 years old) than other New Zealanders (60 years old). This strategic screening policy was implemented as a result of the higher proportion of bowel cancer occurring in Māori before the eligible screening age of 60 years [15].

Other studies [33, 50, 51, 52] have found that males have a higher risk of bowel cancer diagnosis compared to females. This is due to reasons such as the presence of the hormone oestrogen, which is believed to be a protective factor for bowel cancer in females [53], and the fact that the rate of participation in lifestyle‐based risk factors, such as smoking and alcohol use, is higher among males compared with females [54]. However, in the case of early onset, the difference might be roughly similar, and in some studies [10, 20], females have a higher risk of EOBC than males. This study also found that females are 40% more likely to be diagnosed with EOBC than males. However, these findings might also be related to sample size differences and study design.

Rectal cancer is associated with 26% higher odds of being diagnosed with EOBC compared with colon cancer. This result is consistent with the findings of studies conducted elsewhere [10, 33, 55, 56] and may be related to the anatomical position of the rectum often resulting in more noticeable symptoms, such as rectal bleeding and changes in bowel movements, which prompt earlier attention.

While this study has highlighted the emerging public health issue in cancer research by shedding light on racial disparities using realistic administrative data, it has the following limitations: First, the dataset used older clinical data. Nevertheless, clinical data remain crucial for evidence generation, especially considering the long‐term outcomes of diseases and their interventions. Second, health service factors such as general practitioner visits and specialist consultations, and behavioral factors including health‐seeking behaviors, were not included. These will be further investigated in subsequent research as part of a larger project. Third, the study does not consider other important variables that affect survival disparities, such as the stage at diagnosis, which is used as a measure of disease severity. While not identical, we used tumor grade as a proxy indicator for disease severity, despite several unknowns (16.7%) that led to statistically insignificant differences between First Nations and non‐First Nations Australians. Additionally, patients moving elsewhere would not have had their outcomes captured, as the dataset is limited to those who remain in Queensland. Finally, while younger individuals are not generally eligible for the NBCSP, this study is unable to determine how this group has typically been diagnosed. Future studies will use screening data to address this limitation.

## 5 Conclusion

Consistent with the national estimate for Australia, approximately 1 in 10 adult bowel cancer patients in Queensland was diagnosed before the age of 50 years. Young First Nations Australians face more than double the likelihood of a bowel cancer diagnosis compared to young Non‐First Nations Australians. Although younger adults generally have better survival rates of bowel cancer, young First Nations Australians have lower survival outcomes than their Non‐First Nations counterparts.

Therefore, continuing education for health practitioners should include information on the rising incidence of bowel cancer in young people to ensure they consider bowel cancer when consulting younger clients with common symptoms. Given that the current national bowel screening participation rate for First Nations people (50–74 years) is lower than for Non‐First Nations Australians (34.2% vs. 40.5%), future interventions should target younger First Nations Australians who are eligible under the new age threshold [45, 49]. Health promotion initiatives directed at younger First Nations Australians under 45 years old could also help in the early recognition of bowel cancer symptoms. Furthermore, additional research is needed to explore disparities in the diagnosis and survival of bowel cancer among young First Nations and Non‐First Nations Australians. While this study supports the lowering of the bowel screening age eligibility to 45 years, its impact on survival outcomes for First Nations peoples will need to be monitored over time to see if there is a need to further lower the screening age eligibility. This strategy will help to reduce the gap in the bowel cancer burden between First Nations and non‐First Nations Australians.

## Author Contributions

**Tsegaw Amare Baykeda:** conceptualization (lead), formal analysis (lead), methodology (lead), software (lead), writing – original draft (lead), writing – review and editing (lead). **Shafkat Jahan:** conceptualization (equal), methodology (equal), writing – review and editing (equal). **Kirsten Howard:** conceptualization (equal), methodology (equal), writing – review and editing (equal). **Rakhee Raghunandan:** conceptualization (equal), methodology (equal), writing – review and editing (equal). **Gail Garvey:** conceptualization (equal), methodology (equal), supervision (lead), writing – review and editing (equal).

## Ethics Statement

Ethical approval was obtained from the Townsville Hospital and Health Service Human Research Ethics Committee on July 15, 2016 (HREC/16/QTHS/110), the Australian Institute of Health and Welfare (AIHW) ethics Committee on July 15, 2016 (EO2017/1/343), and the University of Queensland Human Research Ethics Committee (UQHREC by 2022/HE002538). Permission to waive consent was obtained under the Public Health Act 2005. De‐identified data are securely uploaded by the AIHW to the Secure Unified Research Environment (SURE) facility, where researchers exclusively access it.

## Conflicts of Interest

The authors declared no conflicts of interest.

## Data Availability

The datasets utilised in this study are not publicly available due to privacy restrictions outlined in our ethics approval, which specifically prohibits data sharing. The corresponding author had complete access to all the data and was responsible for submitting it for publication.
